# Supplementary material for: Identification of photoperiod-induced specific miRNAs in the adrenal glands of Sunite sheep (Ovis aries)
Source: Front Vet Sci. 2022 Jul 22;9:888207. doi: 10.3389/fvets.2022.888207 (PMC9354845; doi:10.3389/fvets.2022.888207)
Supplement: Supplementary file 1 [file Data_Sheet_1.ZIP › Supplementary file/miRDeep2 file.zip!/miRDeep2 file/Know_Structure/SPLP42A3/oar-mir-374b.pdf]

miRBase precursor : oar-mir-374b
Total read count : 13820
oar-miR-374b read count : 13583
remaining reads : 237

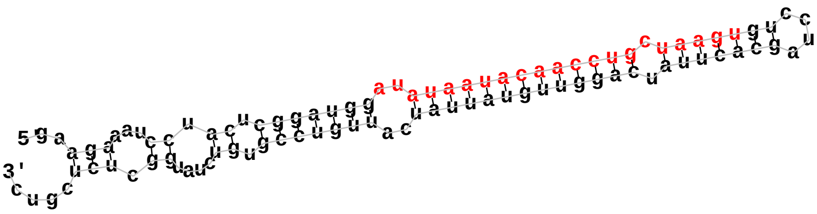

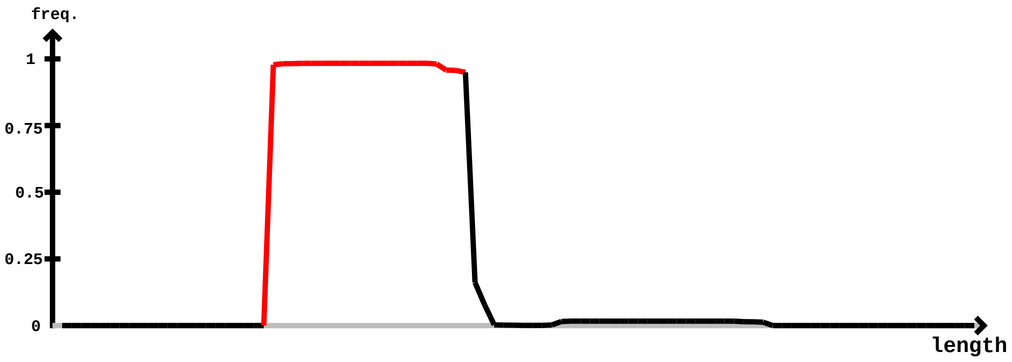


```
                     oar-miR-374b
5'- gaagaaauccuacucggauggauauaauacaaccugcuaaguguccuagcacuuaucagguuguauuaucauuguccgugucuauggcucucguc   -3'    exp
    ((((..((...((.(((((((..(((((((((((((.(((((((....))))))).)))))))))))))..))))))).))..))..)).))...    reads      mm          sample
    .....................auauaauacaaccugAu.........................................................    1          1           seq
    .....................aAauaauacaaccugcu.........................................................    1          1           seq
    .....................auauaauacaaccugcu.........................................................    29         0           seq
    .....................Cuauaauacaaccugcu.........................................................    3          1           seq
    .....................auauaauacaaccugcA.........................................................    2          1           seq
    .....................auauaauacaaccugcua........................................................    318        0           seq
    .....................auauaauacaaccugcuaa.......................................................    13         0           seq
    .....................auauaauacaaccugcuaag......................................................    103        0           seq
    .....................auauaauacaaccugcuaagu.....................................................    10872      0           seq
    .....................auauaauacaaccugcuaagug....................................................    1152       0           seq
    .....................auauaauacaaccugcuaagugu...................................................    1008       0           seq
    .....................auauaauacaaccugcuaaguguc..................................................    10         0           seq
    .....................auauaauacaaccugcuaagugucc.................................................    1          0           seq
    .....................auauaauacaaccugcuaaguguccuagc.............................................    2          0           seq
    .....................auauaauacaaccugcuaaguguccuagcacu..........................................    1          0           seq
    ......................uauaauacaaccugcuaagu.....................................................    15         0           seq
    ......................uauaauacaaccugcuaagug....................................................    3          0           seq
    ......................uauaauacaaccugcuaagugu...................................................    30         0           seq
    ......................uauaauacaaccugcuaaguguc..................................................    3          0           seq
    ......................uauaauacaaccugcuaaguguccuagc.............................................    1          0           seq
    .......................auaauacaaccugcuaagu.....................................................    1          0           seq
    .......................auaauacaaccugcuaagug....................................................    1          0           seq
    .......................auaauacaaccugcuaagugu...................................................    2          0           seq
    .......................auaauacaaccugcuaaguguccu................................................    2          0           seq
    ........................uaauacaaccugcuaagu.....................................................    1          0           seq
    ........................uaauacaaccugcuaagugucc.................................................    2          0           seq
    ........................uaauacaaccugcuaaguguccu................................................    10         0           seq
    ........................uaauacaaccugcuaaguguccuagc.............................................    1          0           seq
    .........................aauacaaccugcuaaguguccuagcacuuauc......................................    1          0           seq
    ..........................auacaaccugcuaaguguccuagcacuu.........................................    1          0           seq
    ............................acaaccugcuaaguguccuagc.............................................    1          0           seq
    .................................................cacuuaucagguuguauuauc.........................    1          0           seq
    .................................................cacuuaucagguuguauuauca........................    1          0           seq
    ..................................................acuuaucagguuguauuauc.........................    18         0           seq
    ..................................................Ccuuaucagguuguauuauc.........................    1          1           seq
```

oar-miR-374b

```
gaagaaauccuacucggauggauauaaauacaaccugcuaaguguccuagcacuuaucagguuguauuaucauuguccgugucuauggcucucguc
...................................................acuuaucagguuguauuauca........................  1    0    seq
...................................................acuuaucagguuguauuaucau.......................  5    0    seq
....................................................cuuaucagguuguauuau..........................  1    0    seq
....................................................cuuaucagguuguauuauc.........................  6    0    seq
....................................................cuuaucagguuguauuauA.........................  2    1    seq
....................................................cuuaucagguuguauuauca........................  3    0    seq
....................................................cuuaucagguuguauuaucau.......................  13   0    seq
....................................................cuuaucagguuguauuaucauu......................  157  0    seq
.....................................................uuaucagguuguauuaucau.......................  1    0    seq
.....................................................uuaucagguuguauuaucauu......................  16   0    seq
.....................................................uuaucagguuguauuaucauug.....................  2    0    seq
......................................................uaucagguuguauuaucauu......................  1    0    seq
```
